# The Impact of Lymphopenia on Delirium in ICU Patients

**DOI:** 10.1371/journal.pone.0126216

**Published:** 2015-05-20

**Authors:** Shigeaki Inoue, Eduard E. Vasilevskis, Pratik P. Pandharipande, Timothy D. Girard, Amy J. Graves, Jennifer Thompson, Ayumi Shintani, E. Wesley Ely

**Affiliations:** 1 Department of Emergency and Critical Care Medicine, Tokai University School of Medicine, Isehara, Kanagawa, Japan; 2 Division of Allergy/Pulmonary/Critical Care Medicine, Department of Medicine, Vanderbilt University School of Medicine, Nashville, Tennessee, United States of America; 3 Department of Anesthesiology, Vanderbilt University School of Medicine, Nashville, Tennessee, United States of America; 4 Department of Biostatistics, Vanderbilt University School of Medicine Nashville, Tennessee, United States of America; 5 Division of General Internal Medicine and Public Health, Department of Medicine, Vanderbilt University School of Medicine, Nashville, Tennessee, United States of America; 6 Veterans Affairs Tennessee Valley Geriatric Research, Education and Clinical Center (GRECC), Vanderbilt University School of Medicine, Nashville, Tennessee, United States of America; D'or Institute of Research and Education, BRAZIL

## Abstract

**Background:**

Immunosuppressed states may predispose patients to development of acute brain injury during times of critical illness. Lymphopenia is a non-specific yet commonly used bedside marker of immunosuppressed states.

**Methods:**

We examined whether lymphopenia would predict development of acute brain dysfunction (delirium and/or coma) in 518 patients enrolled in the Bringing to Light the Risk Factors and Incidence of Neuropsychological Dysfunction in ICU Survivors (BRAIN-ICU) study in medical and surgical ICUs of a tertiary care, university-based medical center. Utilizing proportional odds logistic regression and Cox proportional hazards survival analysis, we assessed the relationship between pre-enrollment lymphocytes and subsequent cognitive outcomes including delirium- and coma-free days (DCFDs) and 30-day mortality.

**Results:**

There were no statistically significant associations between lymphocytes and DCFDs (p = 0.17); additionally, the relationship between lymphocytes and mortality was not statistically significant (p = 0.71). Among 259 patients without history of cancer or diabetes, there was no statistically significant association between lymphocytes and DCFDs (p = 0.07).

**Conclusion:**

lymphopenia, a commonly used bedside marker of immunosuppression, does not appear to be a marker of risk for acute brain injury (delirium/coma) or 30-day mortality in general medical/surgical ICU patients.

## Introduction

Delirium is a neurobehavioral syndrome characterized by fluctuating or abnormal mental status and inattention and an altered level of consciousness or disorganized thinking [1]. Moreover, it is a common complication among patients in the intensive care unit (ICU) [2], and is associated with higher mortality, prolonged length of stay, and long-term cognitive impairment [3,4]. Although several mechanisms have been proposed, including neurotransmitter imbalances, sleep-awake cycle, metabolism, and stress response [5], the pathogenesis of delirium remains unclear.

Recently, cognitive impairment with Alzheimer’s disease, HIV infection, stroke, and dementia have been reported to be related with immunosuppression, characterized by a decreased population and/or dysfunction of lymphocytes[6–10]. For example, a low number of CD4+ lymphocytes is associated with cognitive dysfunction with HIV infection [11]. Immunosuppressive regulatory T cells and expression of programmed death-1, a marker of lymphocyte inactivation, are increased in cognitively impaired patients with Alzheimer’s disease [10]. Furthermore, in animal models of stroke, impaired adaptive immunity, shown by decreased T and B cells, has been associated with increases in pneumonia and nosocomial infections [6,8]. This immunological change, referred to as stroke-induced immunodepression (SIDS), is characterized by (1) lymphopenia, (2) functional deactivation of T helper (Th) cells, and (3) functional deactivation of monocytes [7]. In a mouse brain infraction model, stroke also induced long-lasting lymphopenia with an extensive apoptotic loss of and a shift from T helper cell (Th)1 to Th2 cytokine production in spleen, thymus, and peripheral blood [9].

Beyond central nervous system diseases, lymphopenia and dysfunction of T cells are known predictors of mortality in ICU patients [12]. For example, we previously reported that reduction of immunocompetent T cells and persistent severe lymphopenia (less than 500/μl) may be associated with delayed death after sepsis in elderly patients [13,14]. However, it is not known whether the same relationship exists for the development of acute brain injury in the ICU. As lymphopenia is one of many non-specific yet commonly used bedside markers of immunosuppression, we examined whether lymphopenia was associated with the development of acute brain dysfunction (delirium and/or coma) or 30-day mortality in general medical and surgical intensive care unit (ICU) patients.

## Material and Methods

The institutional review board (IRB) at Vanderbilt University Medical Center, Nashville, Tennessee approved this study. From January 2007 through June 2010, a total of 518 patients admitted to a Vanderbilt University Medical Center medical, surgical, or trauma ICU suffering from delirium were enrolled in the Bringing to Light the Risk Factors and Incidence of Neuropsychological Dysfunction in ICU Survivors (BRAIN-ICU) study, after written informed consent was obtained from the patients or their next of kin. Details of study cohort inclusion and exclusion have been previously reported [15]. Patients were included following admission to a medical or surgical ICU with respiratory failure, severe sepsis, or cardiogenic shock [15]. Exclusion criteria included significant baseline neurological diseases or neurotrauma that would confound the evaluation of delirium. Patients with additional barriers to delirium assessment were excluded, including the inability to understand English, significant hearing loss, moribund patients not expected to survive longer than 24 hours, and persistent coma. We also excluded patients who were not hospitalized at Vanderbilt University because of inaccessibility to the date of lymphocyte counts in these patients.

Data including duration of delirium, severity of illness, comorbidities (including potential immunsuppressive comorbidities), and sedative and analgesic exposures were prospectively collected. The diagnosis of delirium was made using the Confusion Assessment Method for the ICU [16], and daily assessments were made to determine the duration of delirium. The diagnosis of coma was made using the Richmond Agitation Sedation Scale, with a score of -4 to -5 reflecting coma. Outcomes of interest included both mortality and delirium/coma-free days (DCFDs) within the 30-day study period. DCFDs are defined as the number of days alive without delirium or coma during the study period; this measure of acute brain injury takes both coma and mortality into account, rather than focusing only on delirium, which can be misleading.

To consider immune status before delirium and coma, lymphocyte measurements within 2 days prior to BRAIN study enrollment were obtained from electronic medical records at Vanderbilt University Medical Center. If multiple measurements were performed within 2 days before enrollment, we chose the lymphocyte value closest to the enrollment date.

### Statistical analysis

Continuous data are described using medians and interquartile ranges; categorical data are described using frequencies and percents.

To examine the relationship between baseline lymphocyte counts and DCFDs, we used proportional odds logistic regression (POLR); DCFDs has a bimodal distribution, and POLR does not require the assumption of normally distributed errors like linear regression does. We also used robust variance-covariance estimators to produce conservative standard error estimates [17–19]. For associations between baseline lymphocytes and 30-day mortality, we used the Cox proportional hazards model with robust variance-covariance estimators.

Lymphocyte count, which had a skewed distribution, was transformed by taking the cube root in all models in order to prevent undue influence from extreme outliers. Each model included severe sepsis at enrollment as a covariate as well as an interaction term between lymphocyte count and severe sepsis. Previous data demonstrated that sepsis is an important effect modifier of delirium in ICU patients [20]. We adjusted for the following additional covariates, chosen *a priori*, in models with DCFDs as the outcome: age, sex, education, Charlson comorbidity score [21], modified Sequential Organ Failure Assessment (SOFA) score (without a central nervous system component) [22], and receipt of medications between ICU admission and enrollment (benzodiazepines, dexmedetomidine, propofol, and steroids). In Cox regression models assessing mortality, we included only lymphocytes, severe sepsis, and their interaction. Because the model of mortality had a limited effect size, we did not adjust for additional covariates to prevent model overfitting. To account for and reduce potential bias from missing data, we used multiple imputation with predictive mean matching for all models [23].

Because immunomodulatory drugs and conditions may affect the number of lymphocytes [14], we performed sensitivity analyses excluding patients with potentially immunosuppressive states. Our first sensitivity analysis excluded patients with cancer or diabetes. A second sensitivity analysis additionally excluded patients with HIV or with concurrent use of steroids at the time of ICU admission.

Two-sided p-values < 0.05 were considered to be statistically significant. All statistical analyses were performed using R software version 3.0.1 (cran.r-project.org).

## Results

**Fig 1:**
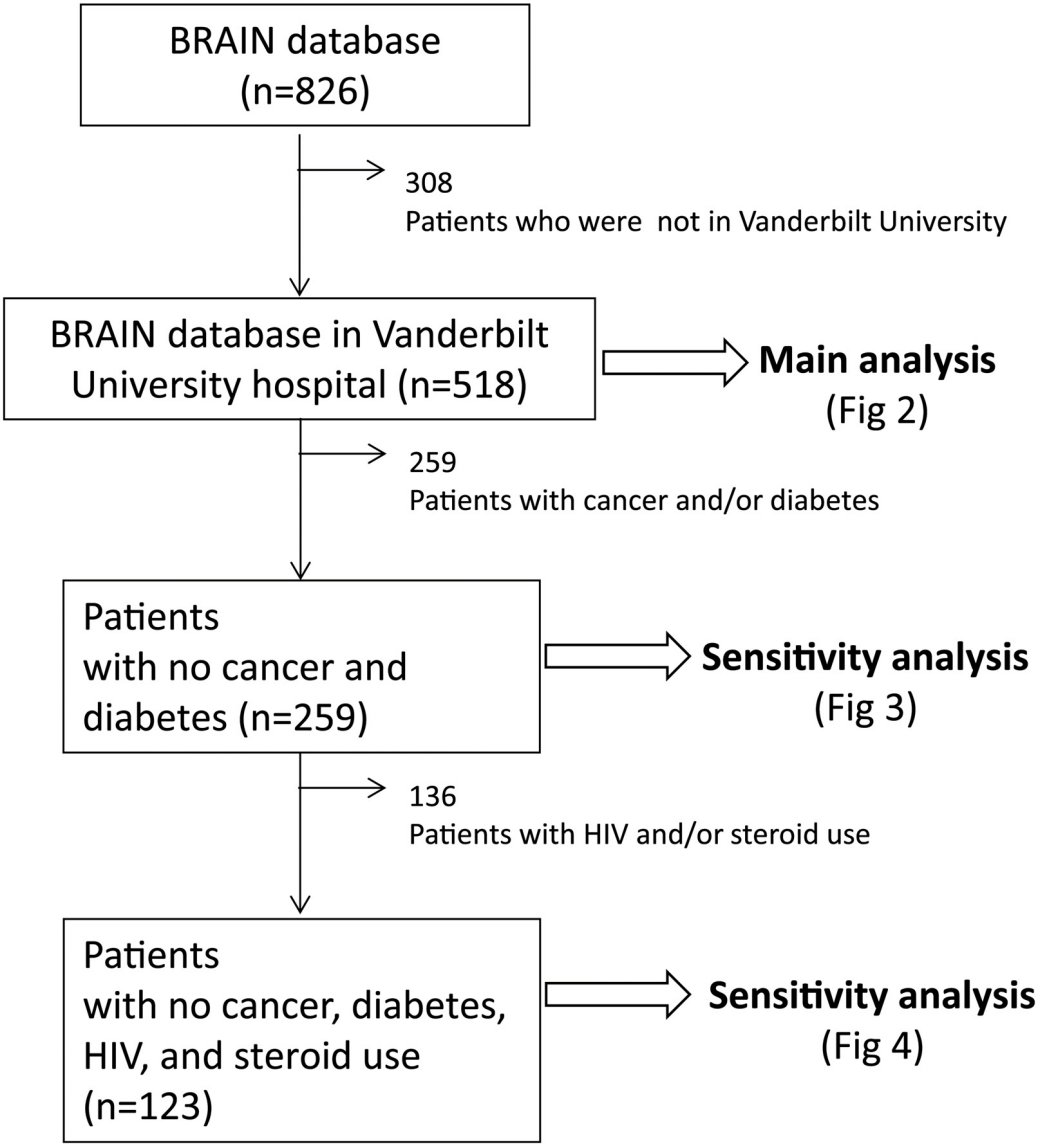
Flow diagram showing two main branching points for patients included in the lymphopenia main analysis and those remaining in the sensitivity analysis.

Of the 518 BRAIN-ICU patients enrolled at VUMC, 259 had no history of cancer or diabetes (Fig 1). Table 1 presents baseline patient characteristics of the study population. Median age at enrollment was 58 (interquartile ratio 47, 68), 52% were male, and Acute Physiology and Chronic Health Evaluation (APACHE) II score at enrollment was 21 (16, 27). Receipt of steroid medications between ICU admission and enrollment was present in 30% of patients. Sedative use prior to enrollment included benzodiazepines in 68%, dexmedetomidine in 4%, and propofol in 28% of patients. Receipt of steroid medication at enrollment was present in 33% of patients. Sedative and opiate use at enrollment included benzodiazepines in 55%, dexmedetomidine in 8%, propofol in 18%, and opiates in 73% of patients. Median DCFDs during the study period was 26 (12, 29) days. Death occurred within 30 days of enrollment in 22% (N = 115) of the population.

**Table 1:** Baseline characteristics of Vanderbilt BRAIN patients.

Characteristic	Vanderbilt BRAIN population (n = 518)	Vanderbilt BRAIN population, no history of cancer or diabetes (n = 259)
Age at enrollment	58 (47, 68)	56 (41, 66)
Sex		
Male	52%	53%
Female	48%	47%
Race		
White	88%	88%
Non-white	12%	12%
Comorbidity		
Cancer	23%	0%
Diabetes	34%	0%
Stroke	7%	6%
Severity of illness		
APACHE II at enrollment	21 (16, 27)	21 (16, 27)
SOFA at enrollment	10 (7, 13)	10 (7, 12)
Sepsis		
Sepsis at enrollment	60%	60%
Severe sepsis at enrollment	58%	58%
Medication		
Receipt of steroid between ICU admission and enrollment	30%	33%
Receipt of benzodiazepine between ICU admission and enrollment	68%	67%
Receipt of dexmedetomidine between ICU admission and enrollment	4%	3%
Receipt of propofol between ICU admission and enrollment	28%	31%
Receipt of steroid at enrollment	33%	36%
Receipt of benzodiazepine at enrollment	55%	55%
Receipt of dexmedetomidine at enrollment	8%	8%
Receipt of opiate at enrollment	73%	71%
Receipt of propofol at enrollment	18%	21%
Hours between ICU admission and study enrollment	26 (17, 48)	24 (16, 45)
Mechanical ventilation		
On mechanical ventilation at enrollment	87%	88%
Length of time on ventilator during ICU stay	3 (1, 8)	3 (1, 7)
Mortality		
30-day mortality during study	22%	18%
Hospital mortality	17%	15%
ICU mortality	15%	13%
Delirium		
Ever delirious during study	74%	71%
Days of delirium during study	2 (0, 5)	2 (0, 5)
Coma		
Ever comatose during study	61%	61%
Days of coma during study	1 (0, 4)	1 (0, 4)
Delirium and coma free days during study	26 (12, 29)	26 (16, 29)
Lymphocyte Count within 2 days of enrollment	0.9 (0.6, 1.5)	0.9 (0.5, 1.6)
Monocyte Count within 2 days of enrollment	0.7 (0.4, 1.1)	0.7 (0.4, 1.1)

Continuous variables presented as median (interquartile range) and categorical variables presented as percent.

### Main analysis

**Fig 2:**
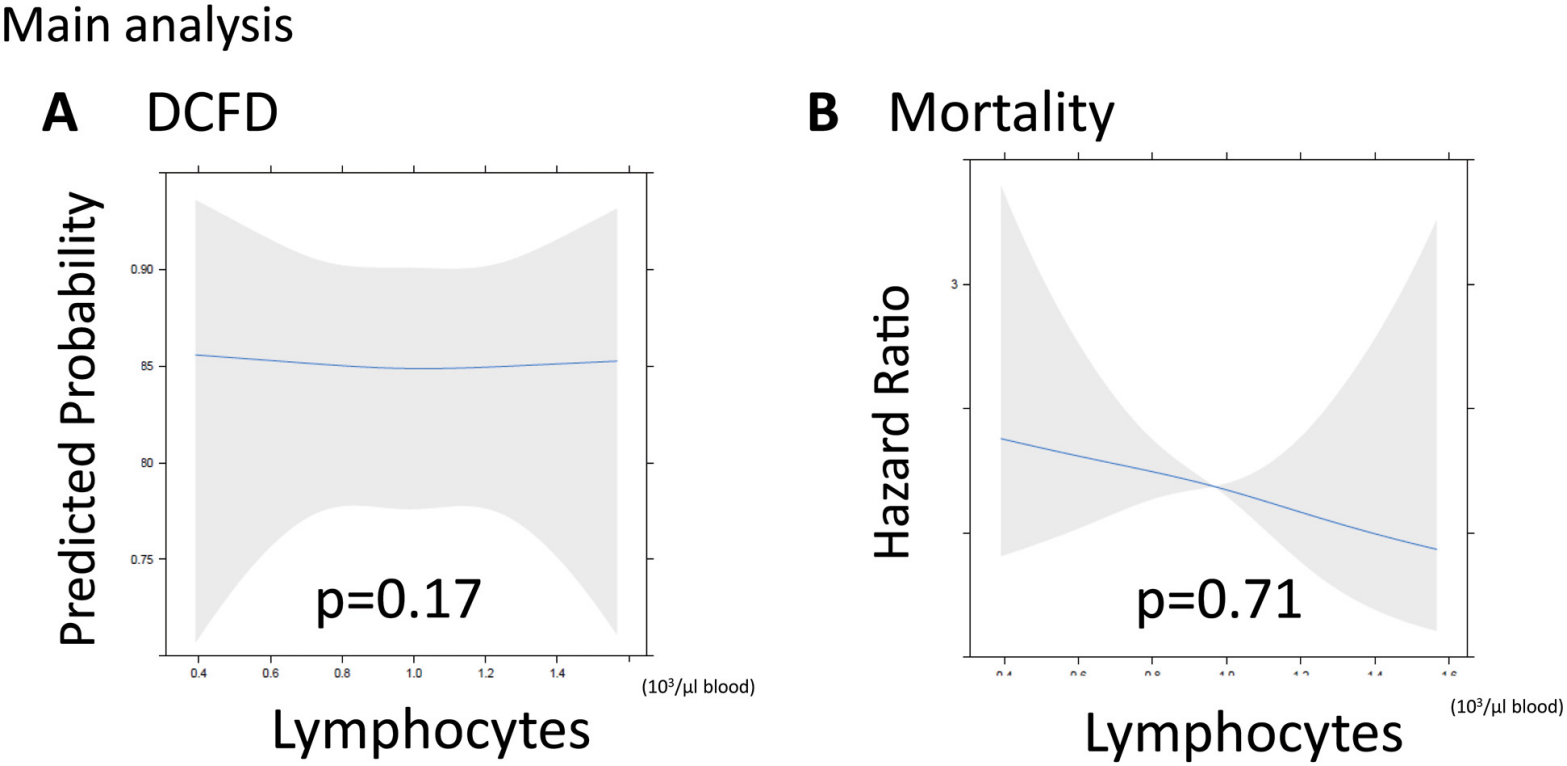
Main analysis: Relationships between lymphopenia and acute brain injury and mortality. **(A)** There was not a statistically significant relationship between lymphopenia and acute brain injury as measured by delirium-free and coma-free days (DCFDs; p = 0.17). DCFDs refer to the number of days patients were alive and free of both delirium and coma in the first 30 days. The unit of lymphocyte count is 10^3^/μL blood. (**B)** Likewise, the hazard ratio between lymphopenia and 30-day mortality was not statistically significant (p = 0.71). The unit of lymphocyte count is 10^3^/μL blood.

There was no statistically significant association between lymphocytes at enrollment and DCFDs after adjusting for covariates (p = 0.17; Fig 2A); this lack of association was true for both septic and non-septic patients (p for interaction = 0.22). There was also no statistically significant association between lymphocytes and mortality after adjusting for covariates (p for lymphocytes = 0.71; p for interaction = 0.70; Fig 2B).

### Sensitivity analysis

**Fig 3:**
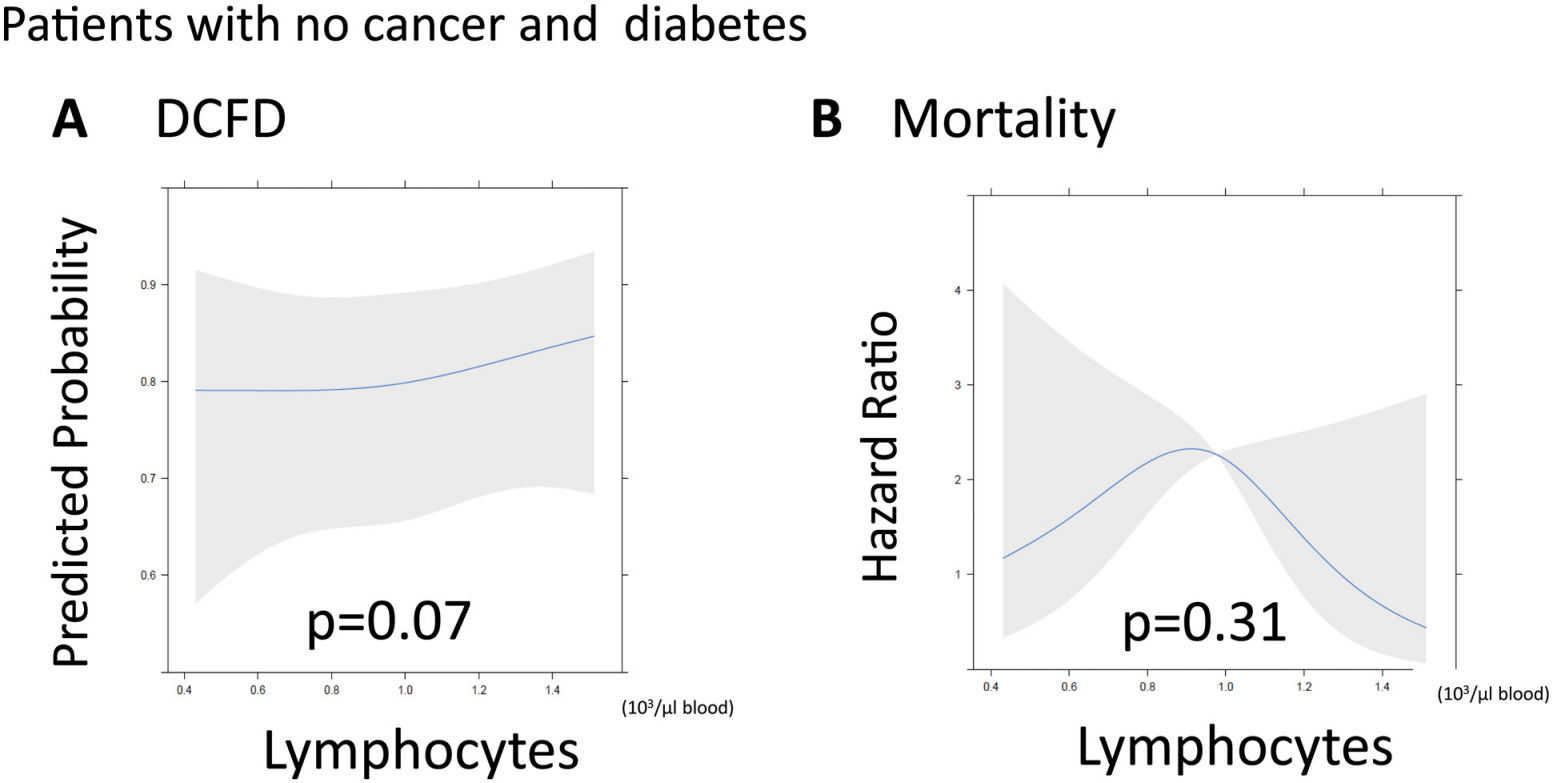
Sensitivity analysis: Relationship between lymphopenia and acute brain injury and mortality of patients without cancer or diabetes. **(A)** There was not a statistically significant relationship between lymphocyte count and acute brain injury as measured with delirium/coma-free days (DCFDs; p = 0.07). DCFDs refer to the number of days patients were alive and free of both delirium and coma in the first 30 days. (**B)** There was not a statistically significant relationship between lymphocyte count and 30-day mortality.

**Fig 4:**
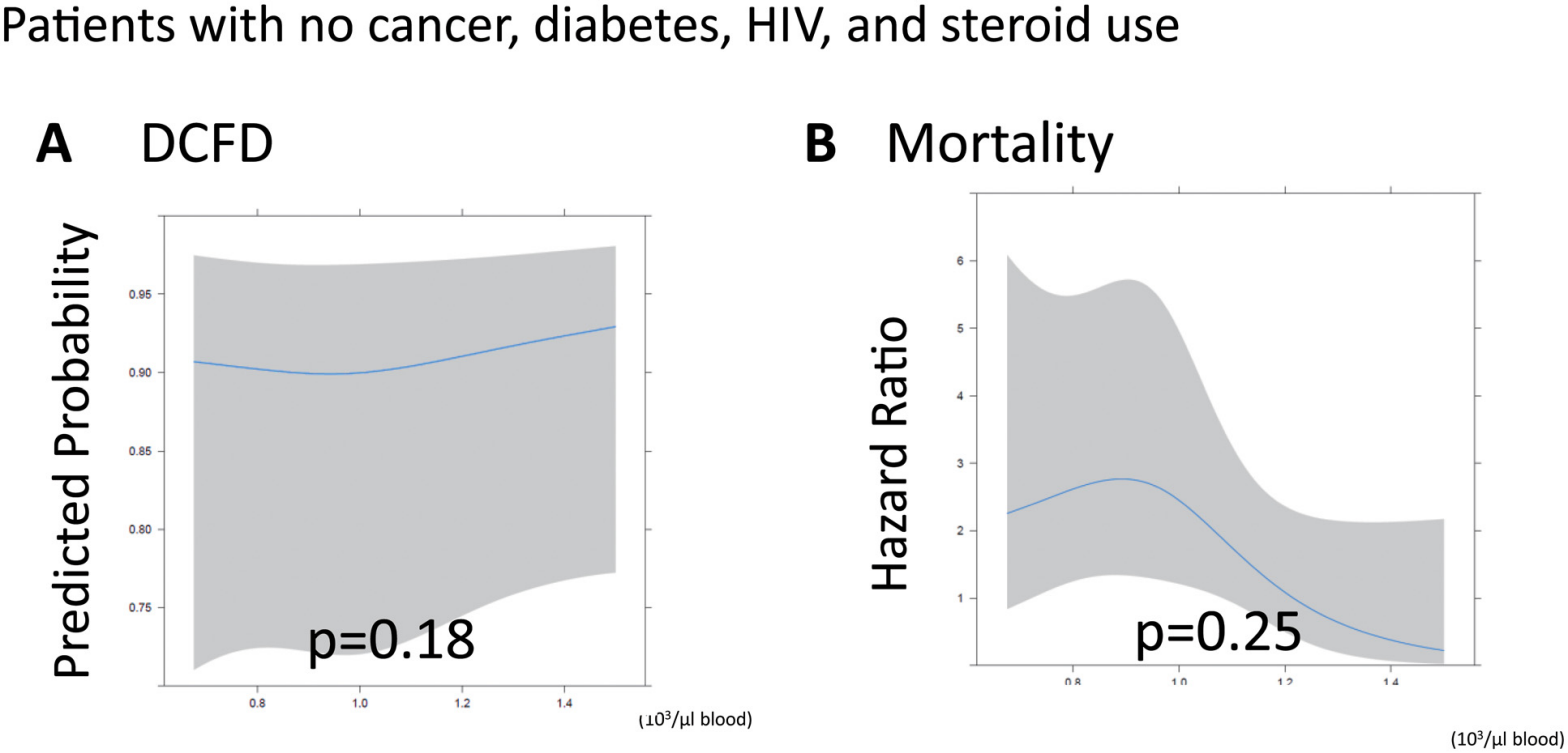
Sensitivity analysis: Relationship between lymphopenia and acute brain injury and mortality of patients without cancer or diabetes, HIV, and steroid use. **(A)** There was not a statistically significant relationship between lymphocyte count and acute brain injury as measured with delirium-free and coma-free days (DCFDs; p = 0.18). DCFDs refer to the number of days patients were alive and free of both delirium and coma in the first 30 days. (**B)** The hazard ratio between lymphopenia and 30-day mortality was not statistically significant (p = 0.25). The unit of lymphocyte count is 10^3^/μL blood. DCFDs refer to the number of days patients were alive and free of both delirium and coma in the first 30 days.

Among the 259 patients without a history of cancer or diabetes, we conducted a sensitivity analysis to determine if those patients had more discernible and potentially significant relationships between our independent and dependent variables. Among patients without history of cancer or diabetes, there were no statistically significant associations between lymphocytes and either DCFDs or 30-day mortality after adjusting for covariates (p = 0.07 and p = 0.31; Fig 3A and 3B, **respectively**). Among patients without history of cancer, diabetes, HIV or steroid use, there was no statistically significant association between lymphocytes and DCFDs or after adjusting for covariates (p = 0.18 and p = 0.25, Fig 4A and 4B, respectively).

## Discussion

There were no significant relationships between lymphopenia and acute brain injury (measured using freedom from coma and delirium via DCFDs) in the current study, suggesting that early hospital lymphocytes levels do not have a significant predictive value with regard to the duration of clinically assessable brain function in ICU patients.

Recently, central nervous system inflammation has been implicated in the development of many neurodegenerative diseases [24,25], suggesting that inflammation may also be related to ICU delirium. Aged mice administered LPS showed delirious behavior with extensive inflammation in the brain [26]. In addition, several observational studies reported associations between the elevation of peripheral inflammatory biomarkers such as interleukin-6, interleukin-8, matrix metalloproteinase-9, and protein C and delirium in ICU patients [27,28].

Alternatively, immunosuppression, characterized by lymphocyte apoptosis, disturbed phagocytosis, and antigen presentation on dysfunctional Alzheimer’s disease s with decreased expression of HLA-DR [29] has been identified as an important cause of mortality in ICU patients [30–34]. Several animal studies have reported that stroke with brain inflammation induces decreased number and function of T and B cells leading to impaired adaptive immunity [6–10]. For example, adoptive transfer of T and natural killer cells from wild-type mice or administration of IFN-γ at day 1 after stroke greatly decreased the bacterial burden [9]. Another study demonstrated that intrastriatal B-cell administration limits infarct size after stroke in B-cell deficient mice, [6]. In light of the evidence linking delirium as an independent risk factor of both in-hospital and long-term mortality [35], we hypothesized that the reduction of peripheral lymphocytes may reflect neuroinflammation and manifest as delirium and coma in ICU patients [36]. Although patients with lower lymphocytes showed a trend towards a higher chance of delirium and coma in the sensitivity analysis (p = 0.07), there was no significant relationship between lymphopenia and 30-day mortality.

There are several potential reasons why lymphopenia was not associated with delirium in ICU patients in the current study. First, the mechanism of inflammation and immunosuppression are possibly different in the central nervous system. Second, lymphopenia represents only one component of immunosuppression, and alternative immunopathological pathways may still have an important role in mediating cognitive dysfunction in the ICU. In addition, we evaluated lymphocyte count but not function. Lymphocyte function, which is characterized by phagocytosis, cytokine production, activation, and expression of co-stimulatory molecules of immune effector cells, remains a topic for a future investigation. Future investigations may also consider the relationship between delirium and dysfunction of immune effector cells. Finally, delirium in the ICU is likely a complicated and multi-factorial process that involves multiple biologic mechanisms.

We did not find a relationship between lymphocytes and 30-day mortality in this cohort, and there are a number of potential explanations for this finding. Previous report showing nonsurvivor in elderly septic patients has lower lymphocytes monitors 90-day mortality, instead of 30-day mortality [14]. Most clinical studies examining patients with sepsis have used 28-day mortality as a clinical end point. However, Winters et al. reports that the use of 28-day or 30-day outcomes in clinical studies may underestimate the morbidity and mortality and may lead to inaccurate inferences because of complications with long-term sequelae, including critical illness, weakness, delirium, and acute lung injury [37]. These results suggest that longer-term outcomes may be more appropriate.

There are important limitations of this study to consider, including generalizability, the outcome specification, the role of immunomodulators, timing of lymphocyte measurements, and missed adjustment of potential confounders. First, this study was conducted in a critically ill population from a single tertiary care center, and results may not generalize less severe ill populations. Second, we chose DCFDs and 30-day mortality as outcome variables, though future studies could explore other relevant outcomes. In addition, a longer observation may be needed to assess the relationship between lymphopenia and outcomes. Third, some immunomodulatory agents such as steroids were administered to patients, which could have confounded our analysis. Fourth, lymphocyte counts were measured within 2 days prior to enrollment. This cutoff was based on investigator opinion, and no standard cutoff currently exists. Alternatively, serial data regarding subtypes of white blood counts may be needed to access the impact of immunity on delirium. Finally, the population of neutrophils was not used in the study, which may offer an alternate pathway of immunomodulation and delirium. Recent research demonstrates neutrophil-to-lymphocyte ratio (NLR) is associated with mortality in a cohort of adult critically ill patients [38]. Despite these limitations, this is the first study to assess the relationship between lymphopenia and acute brain injury and mortality, for which lymphopenia is a measure of immunosuppression frequently used as a bedside indicator of this clinical state.

## Conclusions

Early hospital lymphopenia was not predictive of duration of acute brain injury (delirium/coma) or 30-day mortality in general medical/surgical ICU patients. While it is still possible that this or other immune effector cells may play a role in brain dysfunction, these data do not support that early peripheral lymphocyte measurement will help predict the duration of delirium or coma.

## References

[1] Ely EW, Gautam S, Margolin R, Francis J, May L, Speroff T, et al (2001) The impact of delirium in the intensive care unit on hospital length of stay. Intensive Care Med 27: 1892–1900. 11797025 10.1007/s00134-001-1132-2 PMC7095464

[2] Milbrandt EB, Deppen S, Harrison PL, Shintani AK, Speroff T, Stiles RA, et al (2004) Costs associated with delirium in mechanically ventilated patients. Crit Care Med 32: 955–962. 15071384 10.1097/01.ccm.0000119429.16055.92

[3] Girard TD, Jackson JC, Pandharipande PP, Pun BT, Thompson JL, Shintani AK, et al (2010) Delirium as a predictor of long-term cognitive impairment in survivors of critical illness. Crit Care Med 38: 1513–1520. 10.1097/CCM.0b013e3181e47be1 20473145 PMC3638813

[4] Ely EW, Shintani A, Truman B, Speroff T, Gordon SM, Harrell FE, et al (2004) Delirium as a predictor of mortality in mechanically ventilated patients in the intensive care unit. JAMA 291: 1753–1762. 15082703 10.1001/jama.291.14.1753

[5] Simone MJ, Tan ZS (2011) The role of inflammation in the pathogenesis of delirium and dementia in older adults: a review. CNS Neurosci Ther 17: 506–513. 10.1111/j.1755-5949.2010.00173.x 20553303 PMC6493838

[6] Chen Y, Bodhankar S, Murphy SJ, Vandenbark AA, Alkayed NJ, Offner H, et al (2012) Intrastriatal B-cell administration limits infarct size after stroke in B-cell deficient mice. Metab Brain Dis 27: 487–493. 10.1007/s11011-012-9317-7 22618587 PMC3427715

[7] Hug A, Dalpke A, Wieczorek N, Giese T, Lorenz A, Auffarth G, et al (2009) Infarct volume is a major determiner of post-stroke immune cell function and susceptibility to infection. Stroke 40: 3226–3232. 10.1161/STROKEAHA.109.557967 19661470

[8] Hurn PD, Subramanian S, Parker SM, Afentoulis ME, Kaler LJ, Vandenbark AA, et al (2007) T- and B-cell-deficient mice with experimental stroke have reduced lesion size and inflammation. J Cereb Blood Flow Metab 27: 1798–1805. 17392692 10.1038/sj.jcbfm.9600482 PMC2592689

[9] Prass K, Meisel C, Hoflich C, Braun J, Halle E, Wolf T, et al (2003) Stroke-induced immunodeficiency promotes spontaneous bacterial infections and is mediated by sympathetic activation reversal by poststroke T helper cell type 1-like immunostimulation. J Exp Med 198: 725–736. 12939340 10.1084/jem.20021098 PMC2194193

[10] Saresella M, Calabrese E, Marventano I, Piancone F, Gatti A, Calvo MG, et al (2010) PD1 negative and PD1 positive CD4+ T regulatory cells in mild cognitive impairment and Alzheimer's disease. J Alzheimers Dis 21: 927–938. 10.3233/JAD-2010-091696 20634592

[11] Sacktor N, Nakasujja N, Skolasky RL, Rezapour M, Robertson K, Musisi S, et al (2009) HIV subtype D is associated with dementia, compared with subtype A, in immunosuppressed individuals at risk of cognitive impairment in Kampala, Uganda. Clin Infect Dis 49: 780–786. 10.1086/605284 19622045 PMC2941149

[12] Jimenez-Ibanez EO, Castillejos-Lopez M, Hernandez A, Gorocica P, Alvarado-Vasquez N (2012) High mortality associated with hyperglycemia, neutrophilia, and lymphopenia in critically ill patients. Tohoku J Exp Med 226: 213–220. 22353790 10.1620/tjem.226.213

[13] Inoue S, Suzuki K, Komori Y, Morishita Y, Suzuki-Utsunomiya K, Hozumi K, et al (2014) Persistent inflammation and T cell exhaustion in severe sepsis in the elderly. Crit Care 18: R130 10.1186/cc13941 24962182 PMC4230031

[14] Inoue S, Suzuki-Utsunomiya K, Okada Y, Iida Y, Taira T, Miura N, et al (2013) Reduction of Immunocompetent T Cells Followed by Prolonged Lymphopenia in Severe Sepsis in the Elderly. Crit Care Med 41: 810–819. 10.1097/CCM.0b013e318274645f 23328259

[15] Pandharipande PP, Girard TD, Jackson JC, Morandi A, Thompson JL, Pun BT, et al (2013) Long-term cognitive impairment after critical illness. N Engl J Med 369: 1306–1316. 10.1056/NEJMoa1301372 24088092 PMC3922401

[16] Ely EW, Inouye SK, Bernard GR, Gordon S, Francis J, et al (2001) Delirium in mechanically ventilated patients: validity and reliability of the confusion assessment method for the intensive care unit (CAM-ICU). JAMA 286: 2703–2710. 11730446 10.1001/jama.286.21.2703

[17] Huber PJ (1967) The behavior of maximum likelihood estimates under nonstandard conditions. Proc Fifth Berkeley Symp on Math Statist and Prob 1: 211–233.

[18] White H (1980) A Heteroskedasticity-Consistent Covariance Matrix Estimator and a Direct Test for Heteroskedasticity. Econometrica 48: 817.

[19] White H (1980) Maximum Likelihood Estimation of Misspecified Models. Econometrica 50: 1.

[20] Pandharipande PP, Sanders RD, Girard TD, McGrane S, Thompson JL, Shintani A, et al (2010) Effect of dexmedetomidine versus lorazepam on outcome in patients with sepsis: an a priori-designed analysis of the MENDS randomized controlled trial. Crit Care 14: R38 10.1186/cc8916 20233428 PMC2887145

[21] Charlson ME, Pompei P, Ales KL, MacKenzie CR (1987) A new method of classifying prognostic comorbidity in longitudinal studies: development and validation. J Chronic Dis 40: 373–383. 3558716 10.1016/0021-9681(87)90171-8

[22] Grissom CK, Brown SM, Kuttler KG, Boltax JP, Jones J, Jephson AR, et al (2010) A modified sequential organ failure assessment score for critical care triage. Disaster Med Public Health Prep 4: 277–284. 10.1001/dmp.2010.40 21149228 PMC3811929

[23] Horton NJ, Kleinman KP (2007) Much ado about nothing: A comparison of missing data methods and software to fit incomplete data regression models. Am Stat 61: 79–90. 17401454 10.1198/000313007X172556 PMC1839993

[24] Golde TE (2002) Inflammation takes on Alzheimer disease. Nat Med 8: 936–938. 12205453 10.1038/nm0902-936

[25] Weninger SC, Yankner BA (2001) Inflammation and Alzheimer disease: the good, the bad, and the ugly. Nat Med 7: 527–528. 11329045 10.1038/87839

[26] Godbout JP, Chen J, Abraham J, Richwine AF, Berg BM, Kelley KW, et al (2005) Exaggerated neuroinflammation and sickness behavior in aged mice following activation of the peripheral innate immune system. FASEB J 19: 1329–1331. 15919760 10.1096/fj.05-3776fje

[27] Tsuruta R, Nakahara T, Miyauchi T, Kutsuna S, Ogino Y, Yamamoto T, et al (2010) Prevalence and associated factors for delirium in critically ill patients at a Japanese intensive care unit. Gen Hosp Psychiatry 32: 607–611. 10.1016/j.genhosppsych.2010.09.001 21112452

[28] Girard TD, Ware LB, Bernard GR, Pandharipande PP, Thompson JL, Shintani A, et al (2012) Associations of markers of inflammation and coagulation with delirium during critical illness. Intensive Care Med 38: 1965–1973. 10.1007/s00134-012-2678-x 22903241 PMC3606929

[29] Schefold JC, Hasper D, Reinke P, Monneret G, Volk HD (2008) Consider delayed immunosuppression into the concept of sepsis. Critical Care Medicine 36: 3118 10.1097/CCM.0b013e31818bdd8f 18941324

[30] Hotchkiss RS, Swanson PE, Freeman BD, Tinsley KW, Cobb JP, Matuschak GM, et al (1999) Apoptotic cell death in patients with sepsis, shock, and multiple organ dysfunction. Crit Care Med 27: 1230–1251. 10446814 10.1097/00003246-199907000-00002

[31] Hotchkiss RS, Tinsley KW, Swanson PE, Schmieg RE Jr, Hui JJ, Chang KC, et al (2001) Sepsis-induced apoptosis causes progressive profound depletion of B and CD4+ T lymphocytes in humans. J Immunol 166: 6952–6963. 11359857 10.4049/jimmunol.166.11.6952

[32] Lederer JA, Rodrick ML, Mannick JA (1999) The effects of injury on the adaptive immune response. Shock 11: 153–159. 10188766 10.1097/00024382-199903000-00001

[33] Meakins JL, Pietsch JB, Bubenick O, Kelly R, Rode H, Gordon J, et al (1977) Delayed hypersensitivity: indicator of acquired failure of host defenses in sepsis and trauma. Ann Surg 186: 241–250. 142452 10.1097/00000658-197709000-00002 PMC1396336

[34] Oberholzer A, Oberholzer C, Moldawer LL (2001) Sepsis syndromes: understanding the role of innate and acquired immunity. Shock 16: 83–96. 11508871 10.1097/00024382-200116020-00001

[35] Pisani MA, Kong SY, Kasl SV, Murphy TE, Araujo KL, Van Ness PH (2009) Days of delirium are associated with 1-year mortality in an older intensive care unit population. Am J Respir Crit Care Med 180: 1092–1097. 10.1164/rccm.200904-0537OC 19745202 PMC2784414

[36] Gentile LF, Cuenca AG, Efron PA, Ang D, Bihorac A, McKinley BA, et al (2012) Persistent inflammation and immunosuppression: a common syndrome and new horizon for surgical intensive care. J Trauma Acute Care Surg 72: 1491–1501. 10.1097/TA.0b013e318256e000 22695412 PMC3705923

[37] Winters BD, Eberlein M, Leung J, Needham DM, Pronovost PJ, Sevransky JE (2010) Long-term mortality and quality of life in sepsis: a systematic review. Crit Care Med 38: 1276–1283. 10.1097/CCM.0b013e3181d8cc1d 20308885

[38] Salciccioli JD, Marshall DC, Pimentel M, Santos MD, Pollard T, Celi L, et al (2015) The association between the neutrophil-to-lymphocyte ratio and mortality in critical illness: an observational cohort study. Crit Care 19: 13 10.1186/s13054-014-0731-6 25598149 PMC4344736

